# Investigation of a COVID-19 Outbreak and Its Successful Containment in a Long Term Care Facility in Qatar

**DOI:** 10.3389/fpubh.2021.779410

**Published:** 2021-11-26

**Authors:** Hanadi Al Hamad, Manal Mustafa Mohd. Malkawi, Jameela Ali A. A. Al Ajmi, Mariam Nooh J. H. Al-Mutawa, Sanjay Harish Doiphode, Brijesh Sathian

**Affiliations:** ^1^Geriatrics and Long Term Care Department, Rumailah Hospital, Doha, Qatar; ^2^Rumailah Hospital (RH) & Qatar Rehabilitation Institute (QRI), Doha, Qatar; ^3^NHS-2 National Lead for Healthy Ageing and Focal Point for Elderly in the State of Qatar, Ministry of Public Health, Doha, Qatar; ^4^College of Medicine, Qatar University, Doha, Qatar; ^5^Department of Clinical Medicine, Weill Cornell Medicine-Qatar, Al Rayyan, Qatar; ^6^Hospital Infection Control, Rumailah Hospital, Doha, Qatar; ^7^Corporate Infection Prevention and Control, Quality Management Department, HMC Corporate, Doha, Qatar; ^8^Rumailah Hospital, Doha, Qatar; ^9^Infection Control Committee Rumailah Hospital (RH) & Qatar Rehabilitation Institute (QRI), Doha, Qatar

**Keywords:** COVID-19, elderly, comorbidities, prevention, long term care, outbreak, Qatar

## Abstract

**Introduction:** The objective of this study is to investigate the COVID-19 outbreak and its successful containment in a long-term care facility, Qatar.

**Materials and Methods:** It was a retrospective case series of 24 COVID-19 positive patients inclusive of elderly, patient attenders, and front-liners from 06th to 18th June 2020. Laboratory, radiological, and treatment findings were assessed from electronic records.

**Results:** The outbreak management team concluded that despite all the pre-existing preventive measures implemented at the start of the pandemic, there was still evidence of lapses in infection control practices such as breach of infection control protocols like improper use of personal protective equipment. The infection prevention and control team promptly reassessed and implemented more stringent infection control methods and practices that successfully contained the outbreak on July 1, 2020. Among the seven elderly patients, the average age was 76.28 years ± SD25.5 and all were females. 57% of the patients were symptomatic. The most common comorbidities were Dementia (57%), Diabetes mellitus (43%), Coronary Artery Disease (43%), and Seizures (43%). Ground glass appearances in the lungs were found in 29% of the patients. Among the three deceased patients, Dementia and Coronary Artery Disease were the common comorbidities. Persistent elevation in blood glucose levels was observed among all patients during this period of infection.

**Conclusion:** Elderlies in long-term care facilities are with certain pre-existing comorbidities which makes them more prone to develop COVID-19 complications. Thus, intensive infection control measures like ongoing education and awareness, staff compliance monitoring, quick contact tracing, visitor policy revision, ongoing patient and caregivers monitoring are inevitable recommendations for effective outbreak prevention and management.

## Introduction

The worldwide spread of the Coronavirus was caused by severe acute respiratory syndrome coronavirus 2 (SARS-CoV-2) (1). As of October 06, 2021, nearly 235 million cases of Covid-19 have been reported worldwide, with more than 4.8 million deaths (2). A combination of pharmacologic and non-pharmacological interventions is required to contain its spread. The virus has also had devastating effects on the vulnerable group of our society—people who are 65 years or older—and have pre-existing health conditions (3). Many health conditions that are associated with aging, especially non-communicable diseases such as autoimmune diseases, cancers, heart disease, and metabolic diseases, combined with treatments for these diseases and with immune senescence, substantially affect responses to vaccines and infectious diseases (4). Older people are at high risk of getting infected severely by the virus. Scientific evidence has shown that there is an increase in mortality of Covid-19 patients who are aged more than 50 years (4).

Qatar is one of the world's rapidly developing countries that have a comprehensive healthcare system. It is also dealing with the Covid-19 pandemic impressively. Like the rest of the world, Qatar also had to take strict measures to contain virus spread. The first recorded case of the 2019 Novel Coronavirus (COVID-19) in Qatar was reported on Feb 29, 2020 (5). From then on, the state has instituted measures to contain the viral spread from mass testing of vulnerable groups, including migrant labor workers and nationals returning from overseas, to travel bans, temporary closure of public places, withholding public gatherings, and social distancing methods. In March 2020 (6), it imposed a strict lockdown in the country, limiting the movement of people and urging them to stay home.

Qatar has consistently shown outstanding commitment when it comes to meeting the needs of elderly healthcare; however, while the lockdown and its associated restrictions helped the government to bring down the number of confirmed coronavirus cases, it also created another challenge on how to take care of the elderly given the circumstances introduced by the novel Coronavirus. Particularly, in older patients with certain underlying medical conditions, the virus may cause severe infection, which may delay the recovery of the patient (5). This is especially significant for elderly long-term care patients suffering from multiple comorbidities, as highlighted by the events of an outbreak in a long-term care facility in Qatar, from June 06 to June 18, 2020. This case series within the care facility allowed the opportunity to study transmission events, incubation period, laboratory findings, and treatment and prevention measures.

## Materials and Methods

### Study Design and Participants

The outbreak investigation described in this report was conducted in ENAYA long-term care facility in Qatar. The care facility aptly named ENAYA, an Arabic word meaning long-term, is a specialized care center that works with Hamad Hospital and Rumailah Hospital. It caters to stable patients aged 14 years old and above who require round-the-clock monitoring and supervision from specially trained doctors and nurses. ENAYA is located at the Hamad Medical City and has a capacity of 156 inpatient beds.

This investigation included all the patients, patient attendees, and healthcare workers who were tested positive for the 2019 Novel Coronavirus. The individuals in this report had been linked to a single case of COVID-19 known as the Index Case.

### Contact Classification and Management

On June 7, 2020, the affected facility was informed of the first human case of infection with SARS-CoV-2 in a Somalian national long-term patient in the facility. Between June 7 and June 12, Seven cases of COVID-19 were identified in this cluster. Management and investigation of the outbreak were immediately initiated on June 7 to identify further cases and contacts and understand transmission events and parameters relevant to successful containment, such as hygienic measures, contact tracing, continuous testing and assessment for patients, and attendees, and healthcare workers.

### Case Interviews

An interview was conducted among the identified cases. The interview determined the exact date of symptom onset, identified existing links among cases, established contacts throughout the incubation period. A thorough assessment by physicians and members of the outbreak management team included identifying high-risk cases and contacts, recognizing the presence of comorbidities, and establishing the environment of contact among identified cases. The index case was also thoroughly assessed and interviewed to further establish community links among her and the subsequent cases.

### Laboratory Testing

Laboratory testing involved two swabs (nasopharyngeal and oropharyngeal, pooled) that were stored in a viral transport medium and cooled. RNA was extracted using the QiAamp BioRobot Kit (Qiagen; Hilden, Germany) on a Hamilton Microlab Star as recommended by the manufacturer. Further, real-time reverse transcriptase-PCR (RT-PCR) assays were performed to detect SARS-CoV-2 as per the standard procedure (7).

### Patient and Public Involvement

This research was carried out in response to a national and international public health crisis. There was no involvement from the patients or the public.

## Result

The primary case (Index Case) was a newly hired employee of the long-term health care facility. She developed symptoms of fever (temperature 38.1°C), body pain, and two episodes of vomiting on Jun 6. This prompted her to seek consultation from the staff clinic, where she was tested for COVID-19. On Jun 7, her COVID-19 test came with a positive result and a CT value of 17.44. Incidentally, her husband has been symptomatic since Jun 5, and the screening done on Jun 8 was found to be positive for COVID-19 with a CT value of 24.54. Her husband's diagnosis established the community link between them. Five days prior to her diagnosis, the index case was reported for night duty from 2nd to Jun 3. Consequently, a risk assessment is done by the Infection Control (IC) team, and the UNIT head nurse determined that there was no unprotected exposure; thus, all staff members were considered as low risk. The days following the detection of the Index Case, seven patients together with three patient attendees and 10 other staff members tested positive for the novel Coronavirus.

**Figure 1:**
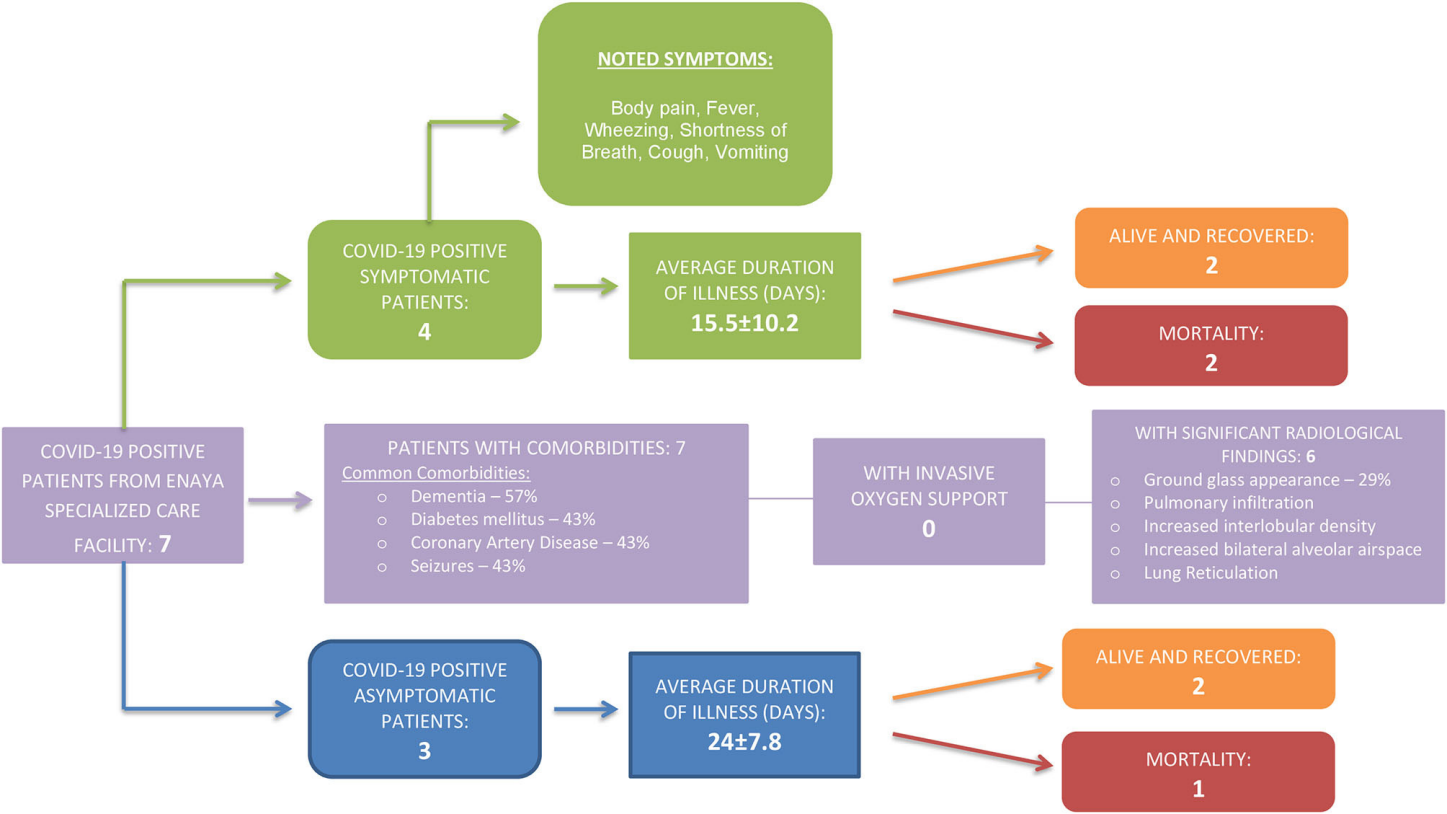
COVID-19 clinical profile of infected long-term care patients.

All seven patients were female with an average age of 76.28 years ± SD25.5. Among these patients, 57% were symptomatic, as illustrated in Figure 1.

**Figure 2:**
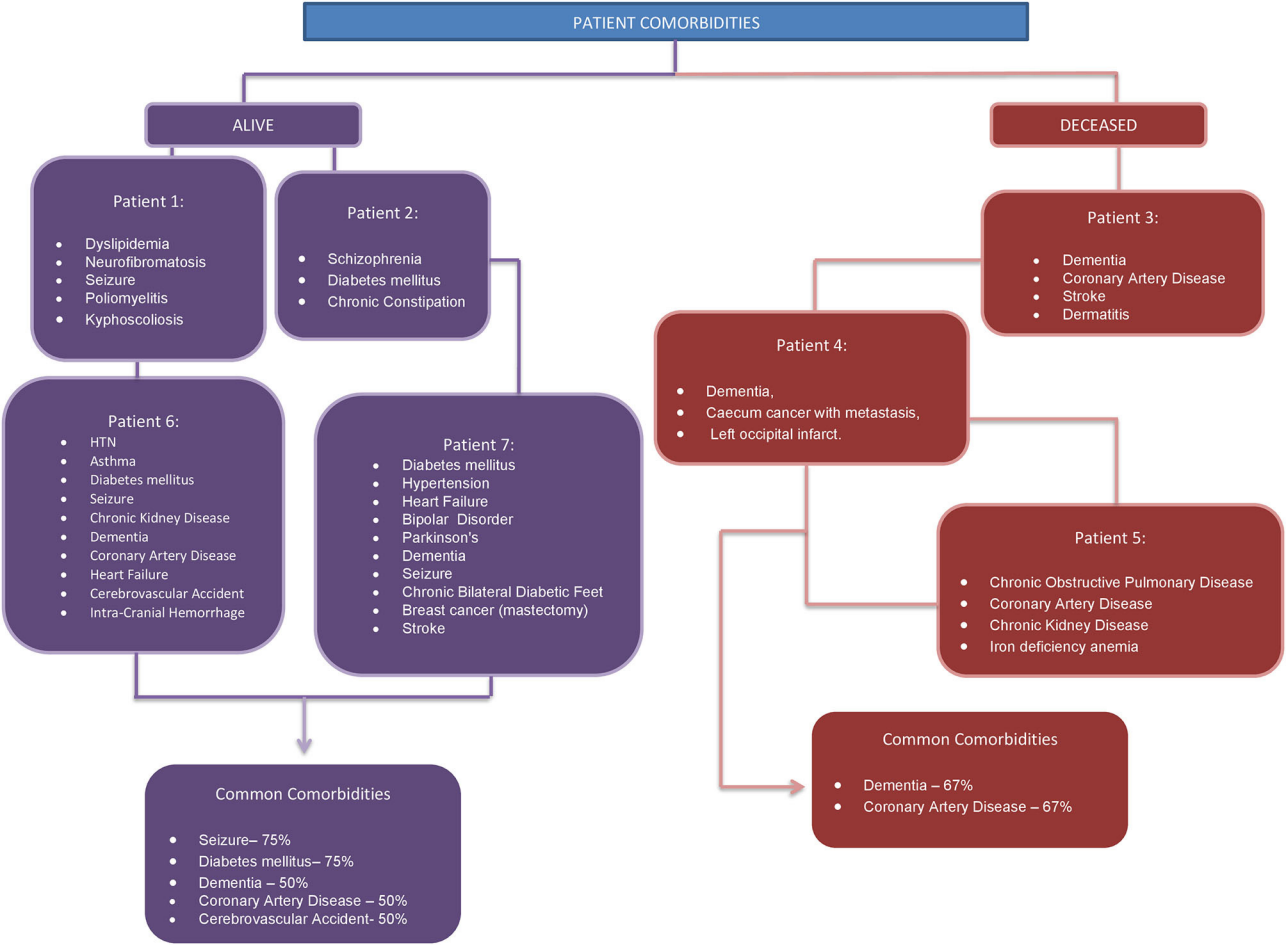
Patient comorbidities.

Most common comorbidities were Dementia (57%), Diabetes mellitus (43%), Coronary Artery Disease (43%), and Seizures (43%) (Figure 2). Ground glass appearances in the lungs were found in 29% of the patients. Among the three deceased patients, Dementia and Coronary Artery Disease were the common comorbidities.

**Figure 3:**
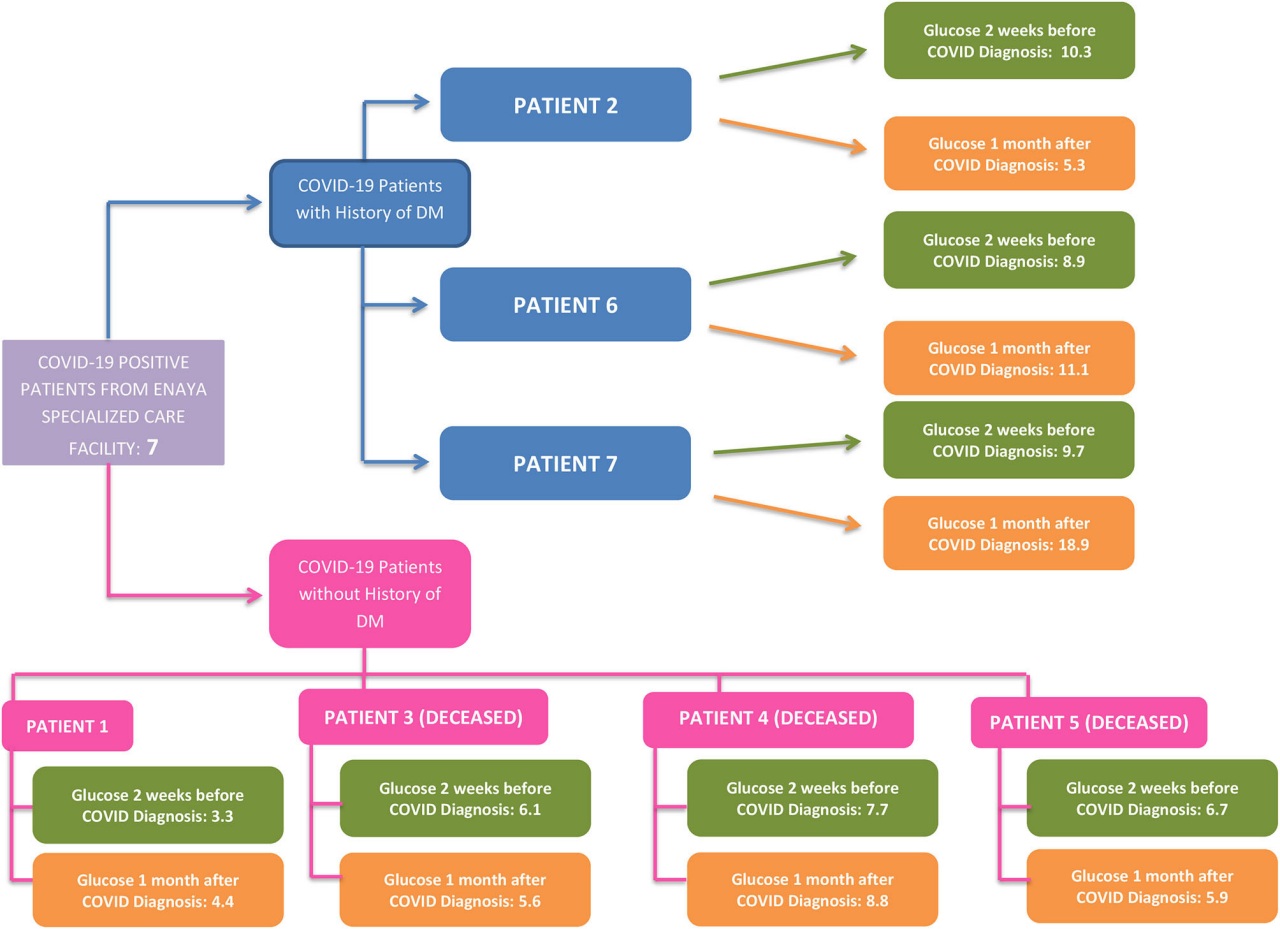
DM history and glucose profile of infected long-term care patients.

Persistent elevation in blood glucose levels was also observed among all patients during this period of infection (Figure 3).

The index case cared for one wheelchair-bound patient (Patient 1) who experienced body pain and headache on Jun 7. Patient 1 was subsequently tested positive for COVID-19 with a CT value of 27.2 on Jun 8. Upon further investigation, it was found out that Patient 1 was non-compliant about wearing the mask on/off and was mobile throughout the unit and conversing with others. Patient 1 is a 47-year-old known to have multiple comorbidities, including Dyslipidemia, Neurofibromatosis, Seizure (Table 1).

**Table 1:** Socio-demographic and clinical profile.

	**Patient 1**	**Patient 2**	**Patient 3**	**Patient 4**	**Patient 5**	**Patient 6**	**Patient 7**
**Demographics**							
Age (In years)	47	40	109	84	101	76	77
Gender	F	F	F	F	F	F	F
Comorbid conditions	Dyslipidemia, neurofibromatosis, seizure, poliomyelitis, kyphoscoliosis	Schizophrenia, type 2 DM, constipation	Dementia, CAD, seborrheic dermatitis scalp, contact dermatitis, stroke	Dementia, caecum cancer with metastasis, left occipital infarct.	COPD, CAD with 2 BMS to LAD, CKD, iron deficiency anemia,	HTN, asthma, DM, seizure, CKD, dementia, CAD, HFrEF (EF: 42%), CVA, intra cranial hemorrhage.	Type 2 DM, HTN, heart failure with EF 30%, bipolar disorder, Parkinson's, dementia, seizure, chronic bilateral diabetic feet, breast cancer (mastectomy), stroke
**Clinical findings**							
Total duration of symptoms prior to admission (days)	0 (Inpatient)	0 (Inpatient)	0 (Inpatient)	0 (Inpatient)	0 (Inpatient)	0 (Inpatient)	0 (Inpatient)
Total duration of illness (days)	21	20 (Reactive)	33(Reactive)	1 (Died)	24	16 (Inconclusive)	19 (Reactive)
Diagnosis	Body pain	Asymptomatic	Asymptomatic	Wheezing	Fever and shortness of breath	Low grade fever, Cough, vomiting	Asymptomatic
Symptoms	Body pain	Asymptomatic	Asymptomatic	Wheezing	Fever and shortness of breath	Low grade fever, Cough, vomiting	Asymptomatic
Temperature (degree Celsius)	36.6	36.4	36.2	36.8	36.2	36.7	36.9
Blood pressure (mmHg)	108/68	106/68	105/60	122/76	110/72	114/54	132/77
Respiratory rate (breaths per minute)	19	18	18	19	18	18	19
Heart rate (Beat per minute)	78	^*^NA	^*^NA	91		78	^*^NA
Chest Xray	^*^NA	Ground-glass appearance of the lungs	Ground-glass appearance of the lungs, diffuse interstitial pattern versus pulmonary fibrosis.		Increased density of interlobular septa and peri-broncho vascular space in relation to linear interstitial pattern	Slight increase in the bilateral mid and lower zones peripheral alveolar airspace opacities with more atelectatic bands	Lung reticulation and interstitial markings
Chest CT	Pulmonary infiltration/ consolidation	^*^NA	^*^NA	^*^NA	^*^NA	^*^NA	^*^NA

* *NA, data Not Available*.

They were admitted under a long-term care facility for a long time, undergoing physiotherapy and occupational therapy, and transferred to the Center for Disease Control (CDC) as a case of asymptomatic COVID-19 infection identified through screening (RT-PCR). SARS-CoV-2 was repeatedly retested as per the protocol at the time, which revealed positive results on repeated examinations for 21 days (Table 2).

**Table 2:** Laboratory Investigations.

**Laboratory investigations**	**Reference range**	**Patient 1**	**Patient 2**	**Patient 3**	**Patient 4**	**Patient 5**	**Patient 6**	**Patient 7**
White blood cells count (x10^3^/μl)	4-10	2.3	2.75	3.37	^*^NA	6.8	8.2	5.1
Hemoglobin (gm/dl)	13–17	13.2	11.9	9.1		9.6	12.3	13.8
Platelet count (x10^3^/μl)	150–400	175	152	153			387	160
Absolute neutrophil count (x10^3^/μl)	2–7	37.7	44.7	63.7		54.5	73.6	68.1
Lymphocyte Count (x10^3^/μl)	1–3	45.6	38.1	21.9		33.7	13.5	16.8
Urea (mmol/L)	3–9	2.7	6.7	NA		14	8.3	2.9
Creatinine (μmol/L)	63.6–110.5	43	85	NA		150	85	50
Sodium	NA	134	144	NA		139	129	3.9
Potassium		3.9	4.4			4.9	5.4	91
Total bilirubin		<2	<3			4	6	3
Albumin		32	38.5			27	32	30
ALT (U/L)	0–55	24	15			6	12	12
AST (U/L)	5–34	39	25			20	18	NA
CK	NA	NA	26	697		NA	NA	
C-reactive protein (mg/l)	0–5	32.1	5	40			8.2	4.1
Influenza A	NA	Negative	NA	NA		NA	NA	NA
Influenza B	NA	Negative						
Mechanical ventilation	No	No	No	No	No	No	No	No

* *NA, data Not Available*.

They were doing fine throughout their stay and were transferred to Rumailah Hospital after being declared cured (Table 3).

**Table 3:** Treatment and outcome.

**Medications**	**Patient 1**	**Patient 2**	**Patient 3**	**Patient 4**	**Patient 5**	**Patient 6**	**Patient 7**
Azithromycin 500 mg daily	No	No	No	No	7 days (10–16/Jun/2020)	No	No
Hydroxychloroquine 400 mg	1 day (08/Jun/2020)	2 days (10th and 16/Jun/2020)	7 days (11th and 13–19/Jun/2020)	No	7 days (11–18/Jun/2020)	4 days (13–17 June 2020)	6 days (13–19 Jun 2020)
Antivirals	No	No	No	No	No	No	No
**Outcome**							
ICU admission	No	No	No	No	No	No	No
Invasive, non-invasive ventilation or supplemental oxygen	No	No	No	No	No	No	No
Duration of symptoms in total (days)	21	20 (reactive)	33 (reactive)	1 (died)	24	16 (inconclusive)	19 (reactive)

On the same day, all patients on the same unit as patient 1 as well as all staff in the facility were screened for COVID-19. Four additional patients (Patient 2-5), two patient attendees, and seven staff tested positive. Patient 2 is a Diabetic, Schizophrenic 40-year-old diagnosed with COVID-19 without any symptoms (Table 1). Investigations were within normal limits, with a Chest X-ray showing the ground-glass appearance of the lungs (Tables 1, 2). They were diagnosed as a case of COVID-19 and transferred to Cuban Hospital for quarantine facility. Their PCR swab showed reactive on the last sample collected on Jun 27, 2020 (Table 3).

Meanwhile, Patient 3 is a 109-year-old known to have multiple comorbidities, including Dementia, CAD, Seborrheic dermatitis scalp, contact dermatitis, was diagnosed COVID-19 without any symptoms (Table 1). Investigations were within normal limits, with Chest X-ray showing ground-glass appearance of the lungs, diffuse interstitial pattern vs. pulmonary fibrosis (Tables 1, 2). They were diagnosed as a case of COVID-19 and transferred to Cuban Hospital for quarantine facility. Their PCR swab showed reactive on the last sample collected on Jul 11, 2020 (Table 3). Similarly, Patient 4 is an 84 year old known to have multiple comorbidities, including Dementia, Caecum cancer with metastasis (Table 1). They were admitted for palliative and end-of-life care. They were having wheezing and were diagnosed with COVID-19 through screening (RT-PCR). They died on the same day due to Cerebral Infarction and antecedent cause's vascular dementia and malignant neoplasm of the rectum. Patient 5, on the other hand, is a 101-year-old known to have multiple comorbidities, including COPD, CAD with 2 BMS to LAD, CKD, Iron deficiency anemia (Table 1). They were admitted to under long-term care facility. They were transferred to acute care as they developed respiratory failure following Respiratory Tract infection (RSV positive) and moved to Cuban Hospital with fever and shortness of breath and diagnosed COVID-19 infection identified through screening (RT-PCR) on 01/Jul/2020. SARS-CoV-2 was repeatedly retested as per the protocol at the time, which revealed positive results on repeated examinations for 24 days (Table 2). They died on 04/Jul/2020 due to Respiratory failure.

Following these events, on Jun 11, the facility's administration decided to keep all positive patients within a single unit (Unit F2) in single rooms with HEPA filters. In contrast, all negative patients were transferred to another vacant unit (Unit G1). Dedicated staff for units F2 and G1 were avoided to be pulled in and pulled out to and from other units. New admissions and therapy activities were also stopped for unit F2, and stricter hygienic measures were also implemented.

It was after the shift to the G1 unit that two more patients tested positive for the virus. Patient 6 is a 76-year-old known to have multiple comorbidities, including HTN, Asthma, DM, Seizure, CKD, Dementia, CAD, HFrEF (EF: 42%) was diagnosed COVID-19 with low-grade fever, Cough, vomiting (Table 1). Investigations were within normal limits, with Chest X-ray showing a slight increase in the bilateral mid and lower zones peripheral alveolar airspace opacities with more atelectatic bands (Tables 1, 2). They were diagnosed as a case of COVID-19 and transferred to CDC for quarantine facility. Their PCR swab showed inconclusive on the last sample collected on Jun 28, 2020 (Table 3).

In contrast, Patient 7 was a 77-year-old also known to have multiple comorbidities, including Type 2 DM, HTN, Heart Failure with EF 30%, Bipolar disorder, Parkinson's, Dementia, Seizure, Chronic Bilateral Diabetic Feet was diagnosed COVID-19 without any symptoms (Table 1). Investigations were within normal limits, with Chest X-ray showing lung reticulation and interstitial markings (Tables 1, 2). They were diagnosed as a case of COVID-19 and transferred to CDC for quarantine facility. Their PCR swab showed reactive on the last sample collected on Jul 1, 2020 (Table 3). The CT values of all seven patients and the patient attendees are detailed (Table 4).

**Table 4:** Records of seven COVID-19 positive patients and three patient attendees.

**Patient no**	**Date of Positivity**	**Result**	**CT value**	**Comments**
1	07-June-2020	+	27.23	Symptomatic with body malaise and pain.
2	08-June-2020	+	19.71	Asymptomatic
3	08-June-2020	+	20.44	
4	08-June-2020	+	14.9	
5	08-June-2020	+	24.75	
6	12-June-2020	+	21.96	Symptomatic after shifting to Unit G1.
7	12-June-2020	+	20.52	
Records of three COVID-19 positive patient attendees				
1	08-June-2020	+	18.55	-
2	08-June-2020	+	18.51	-
3	15-June-2020	+	18.35	-

On Jun 13, the IC chairperson/RH discussed the case scenario with the team to correlate the positive cases and positive staff linkages in light of the staff duty list from May 30 till Jun 6, 2020. The team correlated the CT values and advised and offered to do the sample sequencing of all positive cases, including the husband of the Index case and staff who reported positive on May 30.

The next day, Jun 14, the Head of Virology determined that curtailing the ongoing outbreak is of utmost priority. It recommended the proactive participation of the infection control team and the nursing administration about strict compliance to PPEs and social distancing wherein particular measures are to be taken by patient attendees, including strictly abiding by their specified duties and not indulging in any extra unasked tasks. Additionally, all negative patients, staff, and other healthcare workers were to be screened daily using a soft nasal swab. Respiratory therapists and any other external staff members should be momentarily discontinued, or a team should be dedicated per unit. Sequencing of old positive samples and environmental sampling was also done to extensively understand the viral spread.

Five more staff and one private patient attendant turned positive between 13th to Jun 18, and their CT values and date of positive test result were recorded (Table 5). A total of 23 individuals from the long-term facility center tested positive for the Coronavirus-19, including 13 staff workers, seven patients, and three patient attendees.

**Table 5:** Positive staff with CT values.

	**Date of results**							
	**06/06/2020**	**08/06/2020**	**09/06/2020**	**10/06/2020**	**13/06/2020**	**14/06/2020**	**17/06/2020**	**18/06/2020**
STAFF 1	17.4						29.8	31.6
STAFF 2		18.1					29.8	NEGATIVE
STAFF 3			20.2				24.5	27.4
STAFF 4			26.1		23.7			32.1
STAFF 5			25.4				28.6	36
STAFF 6			27.3				23.8	31
STAFF 7				34.6			23.7	30.4
STAFF 8					26.3		16.8	18.7
STAFF 9					19.1		18.5	22.3
STAFF 10						27.1	16.3	17.7
STAFF 11						38.1	18.8	20.7
STAFF 12								
STAFF 13				NEGATIVE	NEGATIVE	NEGATIVE		19.1

The initial investigation done by the outbreak management team of the facility included in this report revealed the possible causes of transmission, including the breach in the social distancing norms while safety briefing, during endorsement, in medication room and pantry, while giving bath to the patient and near the COW. Additionally, the patient pantry was shared by staff which was also used by patient attendees. Water bottles were also shared between patients, staff, and attendees, whereas eating in the patient pantry also helped in identifying patient trays. Lack of Glove boxes near the patient; hence, HCW reached the nurse station during care to get the gloves. Attendees were carrying gloves in pockets and distributing them to staff whenever needed. Use of personal bags with food material in the pantry, improper way of wearing a surgical mask (Below nose), and removing the mask in between were also identified as possible causes. Aside from this, the outbreak management team also identified the following contributing factors that further spearheaded the viral spread: Non-compliance to the infection control measures even after proper education provided, cross-contamination, frequent close patient-staff contact, movement of the first positive patient, environmental hygiene, improper use of PPE between patients, no social distancing among health care workers, sharing the patient pantry and movement of private patient care attendees among staff population.

Considering the events as an outbreak, an outbreak control team was convened on Jun 16 wherein the team emphasized that external sources, assignment of multidisciplinary teams in multiple units, lack of disinfectants on commonly shared toilets, shared pantry, or in the staff lounge, and lapses in nursing practice, including gathering of suspects during duty or after duty, community exposure and break on social distancing were also probable causes of wide-spread transmission of COVID-19 within the facility despite the strict initial precautions put into place at the beginning of the pandemic.

In addition to the initial measures stated above, an infection control orientation was provided for all healthcare staff after COVID screening (provided they are negative for the same). Mass staff screening, including the non-clinical staff, was performed on the 19th and 20th of June. The second outbreak meeting was convened on Jun 21, where a discussion about false-positive results of the staff and a report on environmental sampling took place. Possible laboratory contamination might have been the cause of the false-positive results of the healthcare workers who had tested positive on initial swabbing while having high CT values and then tested negative on the second swab as confirmed by two different virologists. Meanwhile, report for the environmental sampling revealed positive swab samples from the dirty utility room, consumable store, door knobs/pyxis/automated medication cart, medication carts or handle of medication fridge, housekeeping area, handrails of the hallways, patient room chairs, staff room, air duct system, and within housekeeping areas. The environmental samples were collected from areas around ENAYA, including HEPA air sampling and air duct (HVAC) sampling. Swab samples from surfaces such as mobile phones, doorknobs, toilet seats, computers and keyboards, and patient beds were also collected. Consequently, proper environmental cleaning, particularly for handrails, doorknobs, and handles, and toilet seats, were reinforced. Reeducation of nurses and nursing aides for cleaning medical equipment's surfaces was strengthened, and engineering work for the air duct and filters was also put into action.

Guidelines for symptomatic staff members were also developed. They were instructions for not reporting to work or if at work, to immediately stop patient care activities, compliance to proper donning of personal protective equipment such as surgical masks, reinforcement, and strict adherence to hand and respiratory hygiene and social distancing measures, and finally prompt notification of their supervisor and the infection control team. Moreover, an Infection Control Practitioner performed continuous surveillance to see any breach of infection control practices and monitor compliance to HMC's infection control policies.

Additional directives on infection prevention include the following: Hand Hygiene, Proper Usage of PPE's which includes gloves (no gloves in the corridor) and its proper disposal of PPE's inside patient room, Donning and Doffing of PPE's in a correct way as recommended by IPC team, Social Distancing, Proper cleaning non-critical equipment's like BP apparatus, walkers, wheelchairs, commode chairs and medical equipment's like the pyxis, e-carts, medication carts or bins at the consumable store, Proper Environmental cleaning including door knobs, door handles, compliance in using color-coded cleaning equipment, Strict monitoring the compliance of staff and PA's about the infection prevention and control measures and practices which will be monitored by IPC team and nursing supervisors during after office hours, Housekeeping adherence in infection prevention and control policies including terminal cleaning, disinfection, and do spot-checking.

Finally, on July 1, 2020, the outbreak management team declared the outbreak following no new recorded cases within 14 days of the maximum incubation period.

## Discussion

On Mar 11, 2020, the World Health Organization (WHO) declared the COVID-19 as a pandemic after 118,000 cases of the virus were recorded in over 110 countries and territories all over the world (8). The spectrum of this disease ranges from mild to life-threatening. Some cases might progress rapidly to acute respiratory distress syndrome (ARDS) and/or multiple organ function failure. An epidemiological survey indicated that the general population is susceptible to SARS-CoV-2. Respiratory droplets and contact are considered the main routes of transmission. COVID-19 patients currently remain the primary source of infection. Asymptomatic carriers and those in the incubation period may also be infectious. Recognition, quarantine, and treatment of the confirmed patients are critically important (9).

The seven patients reported in this study were all elderly with accompanying comorbidities. As mentioned by Dai et al. in their study of the effect of comorbidities on elderly patients with COVID-19 (10), comorbidities make medical decisions more complex and challenging, they often involve multiple medications, and the interactions between drugs and diseases often lead to worse final efficacy, worse prognosis, more adverse reactions, and more medical costs. For the management of elderly patients living in long-term healthcare facilities, Bianchetti et al. (11) recommended that monitoring possible contagion among health care professionals should be systematically carried out. The availability and correct use of PPE should be periodically assessed. The geriatric and long-term care of Rumailah hospital recognized that it is critical for the elderly to access emergency and urgent care during the pandemic as such innovations were put in place to help meet the older people's needs, promote their health, and keep them out of the Hospital. These innovations include proactively reaching out to the elderly clients through telephone reassurance, the establishment of dedicated communication channels, videos, and SMS campaigns for the elderly, establishment of quarantine units for patients from abroad, supporting protecting older people living alone in the community, the establishment of national Alzheimer's and memory services helpline, establishment of geriatric telemedicine including consultations and virtual clinics, and establishment of units alternative to ED-urgent and acute care services. Meanwhile, specific approaches designed for long-term care (LTC) patients include implementation of WHO-recommended safety measures that benefit people receiving and providing LTC services, prioritizing testing, tracing and monitoring of COVID-19 among people receiving and providing LTC, identification, and mobilization of staff and resources for appropriate LTC services, scaled-up support for family and caregivers, coordination between services to ensure continuity of care, and securing continued access to care services amidst the pandemic.

Effective management of a COVID-19 confirmed case or outbreak in a facility is dependent upon preparedness. Since the COVID-19 pandemic, several resources have been developed to support facilities in preparing for and responding to outbreaks (12). Similarly, precautionary measures have already been placed within ENAYA following guidelines from the World Health Organization and the Ministry of Public Health. General preventive measures included strict infection control measures in new quarantine units, screening of staff/visitors/ patients at screening desks on entrances for COVID signs and symptoms, implementing and monitoring respiratory and hand hygiene practices, implementing and monitoring social distancing, implementing and appropriate use of PPEs, ensuring food safety, staff education and training, planned staff screening and follow-up, management and surveillance of positive COVID-19 staff as well as patients and staff exposed to COVID-19, terminal cleaning, cancellation of non-essential meetings and educational activities whilst encouraging the use of technology for important meetings, and reallocation of resource as per demand. Meanwhile, specific measures implemented within the facility included a temporary halt inpatient visitation except for end-of-life care patients, video calls as a replacement for physical visits, patient and staff swabbing, halt in gatherings and meetings, implementation of out on pass, reinforced education for staff members, hotel/dorm and transport accommodation for nurses and other staff members.

Strategic implementations and strict compliance to infection control measures augmented with active case monitoring contributed to the immediate containment of the outbreak described in this study. Moreover, sample collection logistics, strict environmental cleaning surveillance, expeditious testing and constant liaising with virology lab, automated method for testing, and adoption of newer environmental collection and testing methods have been the key to the facility's success in containing the outbreak. Still, there is a need for further sequencing of cases. In the investigation of Bohmer and colleagues, they were able to reconstruct and describe transmissions by combining methods of epidemiology and whole-genome sequencing (13).

## Strengths and Limitations of the Study

The strengths of this study include thorough contact tracing partnered with complete assessments and recording with clinical data extracted from the patients' electronic medical records. Moreover, all laboratory procedures and examinations were performed and assessed in one laboratory facility. Infection prevention and management practices have also been discussed thoroughly. However, this investigation can still be improved through additional laboratory findings such as whole genome sequencing that can further describe transmissions and expand available information on the epidemiology of the disease.

## Conclusion

The circumstances surrounding COVID-19 have changed the lifestyle of people of all ages, even more so for patients confined in a long-term care facility and those in the elderly aged group; it is, therefore, essential to create an environment that will support effective care and healthy aging (14). Initial studies of COVID-19 revealed more cases in people 49–55 years of age. Subsequent studies involving more people demonstrated that the prevalence of the disease was higher in individuals ≥60 years of age than in younger individuals (15, 16). In response to this evidence, Hamad Medical Corporation (HMC), in collaboration with the Ministry of Public Health and Primary Health Care Corporation (PHCC), has established a comprehensive plan to help protect this vulnerable group of society. The plan includes comprehensive public education and awareness campaign to inform elderly citizens and their families about adhering to important guidelines for their safety (17). Increasing age limits people's natural immune system to cope with the symptoms of the virus similarly, symptoms are exacerbated when people have additional chronic health condition (18), thus the importance of instituting methods and facilities that will accommodate the needs of the vulnerable. Preventing the introduction of the Coronavirus in these facilities is the most crucial method to avoid an outbreak. Patient and staff assessment and evaluation, rapid screening, access restrictions, protocols for distancing, and astringent hygiene can support this approach.

## Data Availability Statement

The original contributions presented in the study are included in the article, further inquiries can be directed to the corresponding author/s.

## Ethics Statement

The studies involving human participants were reviewed and approved by Medical Research Center (MRC) Ethical Committee at Hamad Medical Corporation, Qatar. Written informed consent for participation was not required for this study in accordance with the national legislation and the institutional requirements.

## Author Contributions

HA designed the study. HA and BS drafted the first version. MM, JA, MA-M, and SD revised the manuscript. All authors contributed to the article and approved the submitted version.

## Conflict of Interest

The authors declare that the research was conducted in the absence of any commercial or financial relationships that could be construed as a potential conflict of interest.

## Publisher's Note

All claims expressed in this article are solely those of the authors and do not necessarily represent those of their affiliated organizations, or those of the publisher, the editors and the reviewers. Any product that may be evaluated in this article, or claim that may be made by its manufacturer, is not guaranteed or endorsed by the publisher.
